# The Awareness of Sudden Infant Death Among Saudi Arabian Women in 2023

**DOI:** 10.7759/cureus.50164

**Published:** 2023-12-08

**Authors:** Sana A Sankari, Amal N AlZaaqi, Haya F AlDawood, Amirah A Alnaser, Lama K Alharbi, Raghad T Zakzouk, Mansour Alqurashi

**Affiliations:** 1 Faculty of Medicine, Almaarefa University, Riyadh, SAU; 2 Department of Medicine and Surgery, Almaarefa University, Riyadh, SAU; 3 Department of Pediatric Cardiology, Almaarefa University, Riyadh, SAU

**Keywords:** mothers’ knowledge, family, cot death, awareness, sudden infant death

## Abstract

Background

Sudden infant death syndrome (SIDS) refers to the unexpected and unexplained death of a child under one year old. The pathogenesis of SIDS remains unclear. However, certain factors such as the child’s sleeping position, sleeping on a soft mattress, and maternal smoking have been suggested to contribute to its occurrence. The objective of this study was to evaluate the level of awareness of SIDS among Saudi Arabian women in 2023.

Methodology

A cross-sectional study was conducted among 300 mothers in Saudi Arabia, over a period of three months, from June to August 2023, using an online questionnaire to gather socio-demographic information from mothers of infants younger than one year old in Saudi Arabia, the sleeping practices of their infants, and their knowledge about SIDS risk factors. Data were coded using the Statistical Package for Social Sciences (SPSS) (IBM SPSS Statistics, Armonk, NY), and statistical significance tests were employed for data analysis. A p-value of <0.05 was considered significant.

Results

Among the 277 participating mothers, 44% were 31-40 years old, 93% were Saudi, 60% were employed, 65% were nonsmokers, and 64% placed babies in a supine position for sleep. About 37% of mothers used a duvet for bedding during summer, compared to 66% who used a duvet during winter, and 81% utilized a soft mattress cover in their children’s beds. Additionally, 67% of mothers reported that their children used a pacifier while sleeping. More than half (54%) of mothers were aware of SIDS with media as their primary source of information. Among those under 21 years old, 50% demonstrated a high level of awareness, compared to 36% of those aged over 50.

Conclusion

Most women in this study were found to be unaware of SIDS. Among those who were aware, the media was the primary source of information. Higher educational attainment was associated with better understanding.

## Introduction

Sudden infant death syndrome (SIDS), or “crib death,” is the unexpected and unexplained death of a child under one year old after all other causes have been ruled out by a comprehensive post-mortem assessment and investigation (e.g., complete autopsy, the examination of the death scene, and the evaluation of circumstances surrounding the infant’s death) [1]. Globally, SIDS is the third most common cause of infant death with a rate of 38.7 deaths per 100,000 live births, and in affluent nations, it is the leading cause of mortality among children aged 1-12 months, with most cases occurring in the first six months of life [2]. Despite ongoing research, the precise etiopathogenesis of SIDS remains uncertain. A combination of factors, including a specific vulnerability, developmental stage, and environmental stressors, may increase the risk [3]. Additional risk factors include sleeping in a prone or side position, sleeping on a soft mattress, excessive bedding, and maternal smoking [4].

There is a significant association between SIDS and maternal substance use, such as alcohol and cannabis, which increases the likelihood of accidental infant suffocation by an intoxicated parent [5]. Indoor air pollution (e.g., from cooking) also has emerged as a relatively recent risk factor for SIDS [5]. SIDS incidence peaks in colder months, when respiratory infections are more prevalent, and deaths tend to occur between midnight and 8 am [1,6]. Regarding SIDS prevention strategies, exclusive breastfeeding for the first six months of an infant’s life and the use of pacifiers are recommended. Immunizing infants can reduce their risk of SIDS by half [6]. However, due to cultural norms and unclear messaging, parents may not adhere to or trust current recommendations, particularly if the advice contradicts their cultural values or lacks clarity. Moreover, in the Kingdom of Saudi Arabia, Islamic beliefs prohibit post-mortem examinations. This barrier to medical and epidemiological studies makes it difficult to assess the rate and impact of SIDS in this population. Thus, it is crucial to understand community perspectives on infant care practices to effectively deliver appropriate messages to target groups [4]. In this study, we aimed to evaluate the awareness of SIDS and its risk factors among Saudi Arabian mothers and to examine whether mothers follow the recommended guidelines for reducing the risk of SIDS.

## Materials and methods

Study group

This study employed a cross-sectional design and was conducted in different Saudi regions, over a period of three months, from June to August 2023. A convenience sampling method was used to recruit a target population of 300 mothers. Eligible participants included mothers of healthy children. We excluded mothers of premature babies and of infants experiencing any of the following medical conditions: apneic episodes, neonatal jaundice, respiratory distress syndrome, cardiac disease, congenital upper airway malformation, central nervous system disorders such as cerebral palsy and developmental delay, or gastrointestinal disorders such as gastroesophageal reflux disease.

Data collection methods and statistical analysis

Data for this study were collected using an online questionnaire distributed via Google Forms (Google, Inc., Mountain View, CA) and shared on various social media platforms. Based on prior studies, primarily by Algwaiz et al. [1], the questionnaire was divided into five parts to gather comprehensive information. The first part collected socio-demographic data from the participants, including their relationship to the child, age, nationality, city of residence, education level, occupational status, and income; the presence of a smoker in the household; and their infant’s age, gender, birth order, birth weight, preterm birth status, and feeding method. The second part of the questionnaire addressed the sleeping practices of the infants, covering topics such as infant sleeping position; the use of a sleep sack or wearable blanket; crib or cot conditions such as the use of pillows, the presence of a cot buffer or bumper, the use of a hard or soft mattress, and the use of a plastic mattress cover; and the thermal environment during sleep such as using heat or air-conditioning. The remaining three sections of the questionnaire assessed the participants’ knowledge of risk factors associated with SIDS, factors related to their awareness of SIDS, and factors influencing their level of knowledge about SIDS, respectively. The collected data were cleared, coded, and entered by using the Statistical Package for Social Sciences (SPSS) (IBM SPSS Statistics, Armonk, NY). The results were presented in tables as frequencies and percentages. Statistical significance tests were used for data analysis. A p-value of <0.05 was considered significant.

Ethical consideration

This study obtained ethical approval from the institutional review board of Almaarefa University under the research project number IRB23-044. Prior to sharing any information, the participants provided their informed consent and could withdraw from the research at any point. Confidentiality was strictly maintained as the participants’ names were not disclosed. Ethical considerations encompassed potential challenges, cultural norms, and legal ramifications to ensure the well-being and rights of the participants.

## Results

Among the 277 mothers who participated from 300 sample members, with 23 participants being excluded due to the exclusion criteria, 44% (n=122) were 31-40 years old. About 93% (n=258) were Saudi, and 66% (n=183) resided in Riyadh. In terms of education and employment, 67% (n=185) had completed college, and 60% (n=166) were employed; 41% (n=114) reported a household income ranging from 10,000 to 20,000 Saudi riyal (SR). Regarding their infants under one year of age, 61% (n=160) were not first-born children. Table 1 summarizes the results.

**Table 1:** Characteristics of the Study Participants

Characteristic	Frequency	Percentage
Age of mother (years)		
<21	10	3.6%
21-30	101	36.5%
31-40	122	44.0%
41-50	33	11.9%
>50	11	4.0%
Nationality		
Saudi	258	93.1%
Non-Saudi	19	6.9%
City of residence		
Riyadh	182	65.7%
Jeddah	35	12.7%
Makkah	9	3.3%
Hail	4	1.4%
Goof	5	1.8%
Medina	4	1.4%
Taif	4	1.4%
Others	34	12.3%
Mother’s education		
Intermediate school and below	8	2.9%
Secondary school	61	22.0%
University	185	66.8%
Postgraduate	23	8.3%
Father’s education		
Intermediate school and below	6	2.2%
Secondary school	27	9.7%
University	177	63.9%
Postgraduate	67	24.2%
Mother’s employment status		
Employed outside the home	167	60.3%
Not employed (housewife)	110	39.7%
Family income		
<10,000	65	23.5%
10,000-20,000	114	41.1%
>20,000	98	35.4%
Relation to child		
Mother	277	91.7%
Father	6	2.0%
Others	19	6.3%
Gender of child		
Male	154	55.6%
Female	123	44.4%
Birth order of child		
First child	107	38.6%
Not the first child	170	61.4%

The participants with more than one child had children ranging in age from under six months to over 40 months, with 34% (n=94) having a child aged 6-12 months (Table 2). The frequency of smoking was highest among fathers at 22% (n=61), and 65% (n=179) reported no smoking in the household (Table 3). It was observed that 64% (n=177) of children slept in a supine position, and 19% (n=53) slept on their side (Table 4).

**Table 2:** Age of Child (Months)

Age of child (months)	Frequency	Percentage
0-5	45	16.3%
6-12	94	33.9%
13-19	53	19.1%
20-26	39	14.1%
27-33	12	4.3%
34-40	8	2.9%
>40	26	9.4%
Total	277	100.0%

**Table 3:** Smoking Status of Parents

Status of smoking	Frequency	Percentage
Father only	61	22.0%
Mother only	12	4.4%
Both parents	25	9.0%
Neither parents	179	64.6%
Total	277	100.0%

**Table 4:** Child Sleeping Position

Child sleeping position	Frequency	Percentage
Prone	47	17.0%
Supine	177	63.9%
Side	53	19.1%
Total	277	100.0%

Regarding safe practices observed by the mothers, 37% (n=104) used a duvet during the summer, compared to 66% (n=184) during the winter; 41% (n=113) put their baby in a sleeping sack or wearable blanket; 60% (n=166) placed a pillow inside the baby’s crib; 63% (n=175) used a cot buffer or bumper; 81% (n=225) used a soft mattress; 47% (n=130) used a plastic mattress cover; and 82% (n=228) used air-conditioning in summer versus 50% (n=137) who used heat in winter. The use of pacifiers during a child’s sleep was reported by 67% (n=186) of the participants, compared to 83% (n=230) who swaddled their infants. Forty-three percent (n=120) reported placing a soft toy in the crib while their baby slept. The practice of co-sleeping with a person other than the parents in the same bed was reported by 43% (n=119), and co-sleeping with a smoker in the same bed was reported by 33% (n=91). Table 5 summarizes the results.

**Table 5:** Sleep Behavior

Sleep practice	Yes	No
Use a bedding duvet during summer	104 (37.5%)	173 (62.5%)
Use a bedding duvet during winter	184 (66.4%)	93 (33.6%)
Use a sleeping sack	113 (40.8%)	164 (59.2%)
Put a pillow inside the baby’s crib	166 (59.9%)	111 (40.1%)
Use a cot buffer	175 (63.2%)	102 (36.8%)
Use a soft mattress	225 (81.2%)	52 (18.8%)
Use a plastic mattress cover	130 (46.9%)	147 (53.1%)
Use cold air-conditioning when the child is sleeping in summer	228 (82.3%)	49 (17.7%)
Use heat when the child is sleeping in winter	137 (49.5%)	140 (50.5%)
Use a pacifier when the child is sleeping	186 (67.1%)	91 (32.9%)
Infant swaddled in general	230 (83.0%)	47 (17.0%)
Soft toy in crib while sleeping	120 (43.3%)	157 (56.7%)
Infant has co-slept with a person (other than the parent) in the same bed	119 (43.0%)	158 (57.0%)
Infant has co-slept with a smoker in the same bed	91 (33%)	186 (67%)

As shown in Table 6, 55% (n=153) of the participants reported that their infants never slept in a separate room from a parent or caregiver. As shown in Table 7, 48% (n=132) reported co-sleeping with their child in the same bed before four month, and 23% (n=64) reported doing so with children older than four months.

**Table 6:** Infant Sleeping Location

Separate room from parents or caregiver	Frequency	Percentage
Yes, before four months of age	58	20.9%
Yes, after four months of age	66	23.9%
No	153	55.2%
Total	277	100.0%

**Table 7:** Infant Co-sleeping

Parents in the same bed	Frequency	Percentage
Yes, before four months of age	132	47.7%
Yes, after four months of age	64	23.1%
No	81	29.2%
Total	277	100.0%

Fifty-four percent (n=150) of the mothers had heard of SIDS from various sources, with 35% (n=53) acquiring information through social media, websites, and written materials and 33% (n=49) obtaining it from healthcare professionals, friends, and relatives (Tables 8, 9). Regarding the level of awareness of SIDS, 31% (n=86) of mothers had excellent awareness, 35% (n=96) had averaged awareness, and 22% (n=61) had poor awareness (Table 10).

**Table 8:** Awareness of Sudden Infant Death Syndrome (SIDS)

Mother heard about SIDS	Frequency	Percentage
Yes	150	54.2%
No	127	45.8%
Total	277	100%

**Table 9:** Source of Information About Sudden Infant Death Syndrome (SIDS)

Source of information about SIDS	Frequency	Percentage
Friend or relative (non-health professional)	45	30.0%
Friend or relative (health professional)	49	32.7%
Social media content, websites, and written information (e.g., books)	53	35.3%
Others	3	2.0%
Total	150	100.0%

**Table 10:** Awareness of Risk Factors of Sudden Infant Death Syndrome (SIDS)

Awareness of risk factors of SIDS	Frequency	Percentage
Excellent	86	31.0%
Average	96	34.7%
Poor	61	22.0%
Not aware	34	12.3%
Total	277	100.0%

Table 11 demonstrates that half (n=5) of the mothers younger than 21 years old had an excellent awareness of SIDS, compared to 35% (n=35) of mothers aged 21-30, 29% (n=36) of those aged 31-40, 18% (n=6) of those aged 41-50, and 36% (n=4) of those older than 50. However, these differences were not statistically significant. As shown in Table 12, 31% (n=51) of employed mothers demonstrated excellent awareness of SIDS, compared to 32% (n=35) of unemployed mothers. Again, this difference was not statistically significant.

**Table 11:** Awareness of Risk Factors of Sudden Infant Death Syndrome (SIDS) by Age

Age (years)	N (%)	Average	Poor	Not aware	Total
<21	5 (50.0%)	2 (20.0%)	0 (0%)	3 (30.0%)	10
21-30	35 (34.7%)	38 (37.6%)	17 (16.8%)	11 (10.9%)	101
31-40	36 (29.5%)	41 (33.6%)	32 (26.2%)	13 (10.7%)	122
41-50	6 (18.2%)	13 (39.4%)	10 (30.3%)	4 (12.1%)	33
>50	4 (36.3%)	2 ​(18.2%)	2 (18.2%)	3 (27.3%)	1
Total	86​ (31.0%)	96​ (34.7%)	61 (22.0%)	34 (12.3%)	277

**Table 12:** Awareness of Risk Factors of Sudden Infant Death Syndrome (SIDS) by Employment Status

Employment status	Excellent awareness	Average awareness	Poor awareness	Not aware	Total
Employed	51 (30.5%)	59 (35.3%)	38 (22.8%)	19 (11.4%)	167
Not employed	35 (31.8%)	37 (33.6%)	23 (20.9%)	15 (13.7%)	110
Total	86 (31.0%)	96 (34.7%)	61 (22.0%)	34 (12.3%)	27

## Discussion

SIDS awareness was found to be statistically related to the participants’ place of residence, with most participants coming from urban areas and only a minority from rural areas. Previous studies similarly have shown a correlation between the knowledge of SIDS and residency location, with rural residents displaying a higher level of SIDS understanding than urban residents [7-9]. However, an alternative study presented a contrasting perspective, indicating that those from urban areas had better knowledge [10]. This discrepancy could be attributed to differences in the traditional knowledge of infant care between rural and urban dwellers.

Additionally, we found that the participants with higher levels of education were more likely to possess superior knowledge of SIDS, but it is important to note that the proportion of participants with advanced educational attainment was relatively small in this particular investigation. Sleeping in a prone position is widely recognized as a significant risk factor for SIDS [11]. Most participants (63.9%) understood that the appropriate sleeping position for infants is supine, compared to 17% who reported placing their babies in a prone position, which is still considerably higher than the 4% incidence of prone sleeping reported in developed countries [11,12]. The use of pillows and soft bedding is risk factors for SIDS, and more than half of the participants in our study reported using them [13,14]. The guidelines established by the American Academy of Pediatrics recommend refraining from placing soft materials and loose bedding in the crib. However, it is important to note that these guidelines do not explicitly discourage the use of blankets [15]. To ensure the safety of infants, wearable blankets or pajamas are recommended instead of loose blankets.

Studies have shown that the combination of bed sharing and maternal smoking greatly increases the likelihood of SIDS [12,16]. Bed sharing was a common practice among mothers in our sample, and 13.4% of mothers were active smokers. These findings highlight the need for intensified efforts to address the awareness of SIDS and its risk factors, even within developed nations. The use of pacifiers as a preventive measure against SIDS remains controversial as pacifier adoption also has been linked to reduced breastfeeding [12,14,17,18]. In our study, a significant number of mothers exclusively breastfed and used pacifiers, despite the potential reduction in breastfeeding frequency associated with pacifier use.

The results of this study indicate that most mothers lack the awareness of SIDS. Those who were aware of SIDS learned about it from media sources. It is crucial for future efforts to strategically engage targeted populations to ensure that information is culturally appropriate and relevant. Media content, websites, and books have been identified as highly effective channels for information dissemination. It is also important to ensure that the dissemination of SIDS information through television and media does not inadvertently exclude socioeconomically disadvantaged individuals. Healthcare professionals play a crucial role in providing education to mothers in this demographic.

Limitations

There are several limitations to our research. First, the findings are derived from a single nation and thus may not accurately represent the global or broader population. Conducting the study within a single nation limits the generalizability of the results, as the sample of participants and specific context may not fully capture the diversity and complexities present in other countries or regions. Future studies should aim to include participants from a wider range of geographic locations to enhance the external validity of the findings.

Furthermore, it is important to acknowledge the limitations in assessing genetic and viral risk factors for SIDS. Due to various constraints, we were unable to fully evaluate these factors in our research. This limitation highlights the need for additional studies to provide a more precise assessment of the prevalence of SIDS and other risk factors among mothers who have experienced the loss of a child to suspected SIDS.

## Conclusions

Our study revealed a significant lack of knowledge regarding SIDS among mothers. Media sources were the primary means through which women became aware of SIDS. The participants with higher educational attainment demonstrated a greater understanding of SIDS. Furthermore, the statistical connection between the awareness of SIDS and the place of residence was evident, with most participants residing in metropolitan regions and a small percentage from rural areas.

The study results show that many mothers know that babies should be placed on their backs to sleep. Additionally, a significant number of participants in our study recognized the potential risks posed by the use of pillows and soft bedding in relation to SIDS. Bed sharing was a common practice among the mothers in our sample, further highlighting the need for awareness and education regarding safe sleep practices. To gain a better understanding of the relationship between pacifier use, maternal smoking, and SIDS, further research is warranted.
